# CD117/c-kit in Cancer Stem Cell-Mediated Progression and Therapeutic Resistance

**DOI:** 10.3390/biomedicines6010031

**Published:** 2018-03-08

**Authors:** Brittni M. Foster, Danish Zaidi, Tyler R. Young, Mary E. Mobley, Bethany A. Kerr

**Affiliations:** 1 Department of Cancer Biology, Wake Forest University School of Medicine, Winston-Salem, NC 27157, USA; bmfoster@wakehealth.edu (B.M.F.); dzaidi@wakehealth.edu (D.Z.); tyler.r.young222@gmail.com (T.R.Y.); mmobley@wakehealth.edu (M.E.M.); 2 Wake Forest Baptist Comprehensive Cancer Center, Winston-Salem, NC 27157, USA

**Keywords:** CD117/c-kit, cancer progression, cancer stem cell, tumor-initiating cell, metastasis, tyrosine kinase inhibitor, stem cell factor

## Abstract

Metastasis is the primary cause of cancer patient morbidity and mortality, but due to persisting gaps in our knowledge, it remains untreatable. Metastases often occur as patient tumors progress or recur after initial therapy. Tumor recurrence at the primary site may be driven by a cancer stem-like cell or tumor progenitor cell, while recurrence at a secondary site is driven by metastatic cancer stem cells or metastasis-initiating cells. Ongoing efforts are aimed at identifying and characterizing these stem-like cells driving recurrence and metastasis. One potential marker for the cancer stem-like cell subpopulation is CD117/c-kit, a tyrosine kinase receptor associated with cancer progression and normal stem cell maintenance. Further, activation of CD117 by its ligand stem cell factor (SCF; kit ligand) in the progenitor cell niche stimulates several signaling pathways driving proliferation, survival, and migration. This review examines evidence that the SCF/CD117 signaling axis may contribute to the control of cancer progression through the regulation of stemness and resistance to tyrosine kinase inhibitors.

## 1. Introduction

When diagnosed early, primary tumors can be treated, and in some cases, the cancer can be considered cured. A subset of patients will experience a recurrence of the primary tumor in the same site, and it is hypothesized that this is due to remaining therapeutic resistant cells, called cancer stem cells (CSCs). The CSC theory postulates that a subpopulation of tumor cells remaining after resection drive recurrence, while tumor cells surviving the circulation and arresting at metastatic sites driving tumor growth are metastatic CSCs [1]. In either case, CSCs are capable of self-renewal and asymmetric division and may be able to recapitulate the initial tumor heterogeneity. Further, these CSCs are more resistant to most treatments [1,2,3,4,5,6,7,8].

Cancer progression and therapeutic resistance are directly related to metastasis, the main cause of cancer-related death. Currently, there are no interventions to prevent metastasis or, in many cases, to treat the metastatic tumor. Thus, there is a need to understand how cells enter and survive the circulation, and then develop into overt metastases in another niche or home. One current hypothesis is that a subset of tumor cells control metastasis. While in the circulation, these metastatic cells are called circulating tumor cells (CTCs), and when in the metastatic niche, disseminated tumor cells (DTCs). Although approximately 3.2 × 10^6^ cells/g tissue are shed from tumors daily, <0.01% develop into metastases [9,10]. Thus, not all CTCs and DTCs can form a micro- or macrometastases, as many cells remain dormant within the metastatic tissue, and many do not survive the shear stresses, oxygen tension changes, and other stressors in the circulation. Growth of the metastatic tumor and recapitulation of the primary tumor heterogeneity in a secondary site are driven by metastatic CSCs [11,12]. Asymmetric division of CSCs allows for the maintenance of the CSC population, as well as expansion of cells representing the full spectrum of the original tumor. Several markers for CTCs and CSCs have been postulated in the literature [13]. We and others have demonstrated that CD117 is expressed in aggressive cancers, on CTCs, and in recurrent and resistant tumors [14,15,16,17]. This review will examine the evidence that CD117 and its activation in CSCs may contribute to the control of tumor progression and therapeutic resistance.

## 2. The CD117 Receptor

The CD117 gene, officially known as “KIT proto-oncogene receptor tyrosine kinase” (GenBank ID: 3815), is also more commonly known as c-kit, kit, or stem cell factor receptor. CD117 was first identified as the cellular homolog of the feline sarcoma viral oncogene v-kit. The CD117 gene consists of a single copy located on chromosome 4 (4q12) encompassing ~88 kb (base pairs 54,657,927 to 54,740,714) and spanning twenty-one exons producing a transcript of 5.23 kb [18,19,20]. The cDNA of CD117 encodes a 976 amino acid protein of 145 kDa [21,22]. The resultant CD117 protein is a member of the type III receptor tyrosine kinase family, which also includes CSF-1R, PDGFRβ, PDGFRα, and FLT3 [23,24]. This receptor tyrosine kinase family is defined by an extracellular domain with five immunoglobulin-like loops, a highly hydrophobic transmembrane domain (23 amino acids for CD117), a juxtamembrane domain, and an intracellular domain with tyrosine kinase activity split by a kinase insert in an ATP-binding region and in the phosphotransferase domain [24,25,26,27,28]. The CD117 protein contains ten known glycosylation sites and is largely conserved between species, with the human protein having ~83% homology to mouse and ~68% homology to chicken [29]. CD117 and the other type III receptor tyrosine kinases are an important piece in cell signaling and are responsible for maintaining cell functions such as cell survival, metabolism, cell growth and progression, proliferation, apoptosis, cell migration, and cell differentiation [30,31,32]. These are important in understanding the biology of cancer cells.

### 2.1. CD117 Splice Variants

It has been demonstrated that CD117 of both mice and humans is expressed as two different isoforms, caused by alternative splicing, with only four amino acids differing (glycine, asparagine, asparagine, lysine, abbreviated as GNNK). These amino acids are either present or absent upstream of CD117’s transmembrane domain (GNNK+ GenBank ID: NM_000222 and GNNK− GenBank ID: NM_00109372 [33,34], with respective sizes of 5190 and 5178 bp). Several studies demonstrated that these splice variants, depending on the cell type, can activate different signal transduction pathways and their effects on tumorigenicity, confer constitutive tyrosine phosphorylation, and stimulate association with phosphatidylinositol 3-kinase (PI3-K) [35,36]. In 1999, a study demonstrated that isoform GNNK− transformed NIH3T2 fibroblasts caused tumorigenicity in nude mice [37]. Another study from 2003 showed increased expression of the GNNK− isoform in testicular germline cell tumors, compared to the normal testis which had a higher expression of GNNK+ CD117 receptor [38]. While GNNK− has a higher affinity for SCF, CD117’s ligand, as well as faster phosphorylation kinetics, the GNNK− isoform is the dominant isoform in normal tissue, such as bone marrow and melanocytes. Other studies suggest the ratio of GNNK−/GNNK+ is what causes tumorgenicity, with a higher ratio of GNNK−/GNNK+ being the driving force when the D816V mutation is present [39]. Additional studies are required to understand the physiological and oncogenic roles of these isoforms.

### 2.2. Common CD117 Oncogenic Mutations

CD117 develops an overactivation or ligand-independent constitutive mutation to become oncogenic. Overactivation of CD117 causes alterations in the signaling pathways upregulating proliferation, cell survival, migration, and differentiation. Gain of function mutations have been linked to several malignancies, including acute myeloid leukemia, gastrointestinal stromal tumor, mast cell leukemia, melanoma, and testicular cancer [40]. These mutations are shown to occur in the tyrosine kinase domain 1 (TK1, exon 17) and the juxtadomain region (JM, exon 11). Less common mutations occur in the extracellular domain (exons 2, 8, and 9), as well as tyrosine kinase domain 2 (TK2, exons 13, and 14) [41]. These mutations can occur in a variety of ways, such as point mutations, frame deletions, and internal tandem repeats, but rarely do we find more than one mutation of CD117 in tumors. A list of mutations is further reviewed elsewhere [39,42,43].

## 3. CD117 Expression in Normal Stem Cells

**Figure 1:**
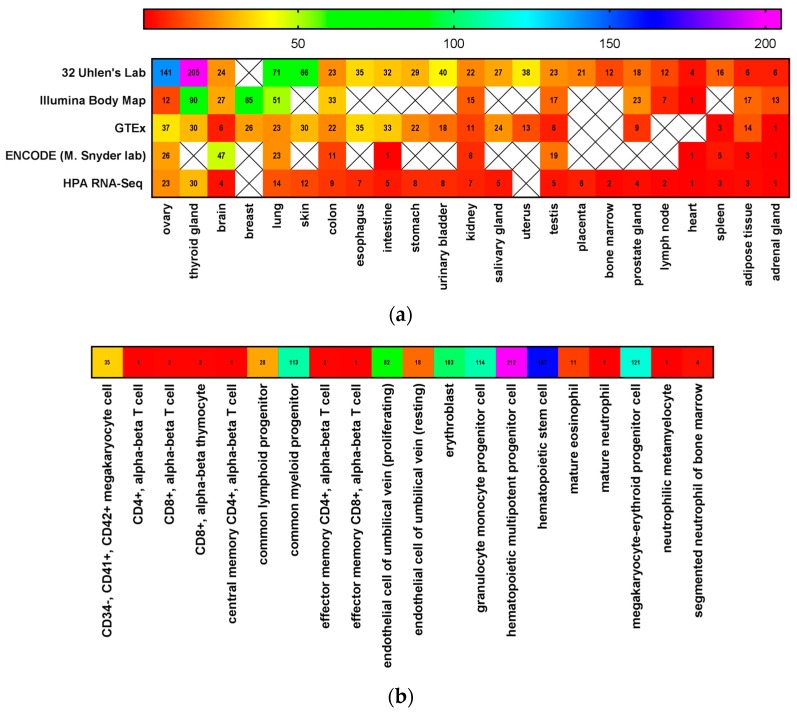
CD117 is expressed in normal tissues. CD117 expression in (**a**) normal tissues and (**b**) bone marrow progenitor cells using data mined from the EMBL—European Bioinformatics Institute Gene Expression Atlas [53] and the NIH GenBank [33,34,54].

Stem cells are defined by the National Institutes of Health as those that can divide for an indefinite period of time to develop specialized cells and organs [44]. These cells possess an ability to continuously self-renew and differentiate into unique cell types based upon their progenitor cells, allowing for tissue homeostasis and regeneration [45,46]. This is made possible by asymmetrical cell division, whereby one daughter cell is identical to its mother, and the other daughter cell has continued potential for differentiation [47,48,49]. Stem cells can be identified by certain markers, such as CD133, CD44, CD34, and CD117 [50,51]. The expression of CD117 in tissues and stem cell niches are shown in Figure 1. CD117 is expressed, for example, in stem cells in the murine prostate. A single CD117 positive cell, which was also lineage-negative and expressed Sca-1, CD133, and CD44, regenerated an entire secreting prostate when mixed with urogenital mesenchymal cells and implanted on the renal capsule. Thus, this CD117-expressing cell was considered a prostate stem cell in adult tissue [52]. While each tissue contains a subpopulation of stem cells, the largest reservoir of stem cells in the body is the bone marrow [51].

Within the bone marrow, there are several stem cell populations, but most prevalent are hematopoietic stem cells (HSCs) [55,56]. HSCs are pluripotent cells defined by their ability to proliferate and self-renew into all of the hematopoietic cell lineages throughout the organism’s lifetime [57]. These cells can also differentiate into endothelial cells [58]. CD117 plays a key role in the HSC stemness, such as the ability to proliferate and differentiate [59]. Immature HSCs express CD34 in addition to CD117. As the cells mature and differentiate, they begin to lose the expression of CD117 along with their stemness.

Outside the bone marrow, CD117 is required for hematopoiesis in the spleen and liver niches. CD117 deletion in the spleen or bone marrow leads to a loss of the lymphocyte and erythrocyte lineages, while platelet numbers remain the same [60]. Thus, CD117 expression is required for several branches of hematopoietic cell differentiation.

## 4. SCF Expression in Stem Cell Niches

CD117’s sole ligand SCF (GenBank ID: 4254 [33,61]), also known as mast cell growth factor, kit ligand (KL), or steel factor, is a hematopoietic cytokine derived from bone marrow that is widely expressed [58,62]. This ligand is a glycosylated, non-covalent homodimer, and is expressed at variable concentrations throughout the body. SCF exists either as a soluble secreted form (NM_000899 at 5376 bp) or a membrane-bound form (NM_003994 at 5460 bp) depending on whether the region containing exon 6 is spliced, which leads to the released soluble form [27,55,63]. Both isoforms are bioactive but vary in their effectiveness in activating CD117 [64].

SCF plays a vital role in stimulating mature and primitive HSCs maintaining survival, promoting proliferation, and regulating growth and development of HSCs [16,22,27,65]. SCF is expressed in niche cells controlling CD117 positive HSCs from mid-gestation through adulthood [66]. Bone marrow niche cells secreting SCF include perivascular cells, endothelial cells, pericytes, mesenchymal stem cells, megakaryocytes, and stromal cells [67,68]. Additionally, osteoblasts produce SCF and control CD117-expressing HSC numbers near trabeculae [69]. Furthermore, osteocytes, chondrocytes, and adipocytes differentiating from mesenchymal stem cells also express SCF [70]. SCF is secreted by megakaryocytes and osteoblasts and is capable of enhancing the differentiation of megakaryocytes and osteoclasts [71]. SCF deletion in endothelial cells or pericytes leads to HSC depletion in bone marrow [67,70].

Outside the bone marrow, SCF is expressed in the spleen and liver to support extramedullary hematopoiesis [72,73]. Within the spleen, SCF is produced by red pulp endothelial cells and perivascular stromal cells, and in the white pulp by central arteriolar cells and rare stromal cells. Extramedullary hematopoiesis increased the numbers of these SCF-expressing cells throughout the spleen. However, the CD117 positive HSCs were only located in the red pulp of the normal spleen [72]. Thus, SCF controls CD117 induced cell mobilization and homing to stem cell niches.

## 5. CD117 Activated Signaling Pathways

**Figure 2:**
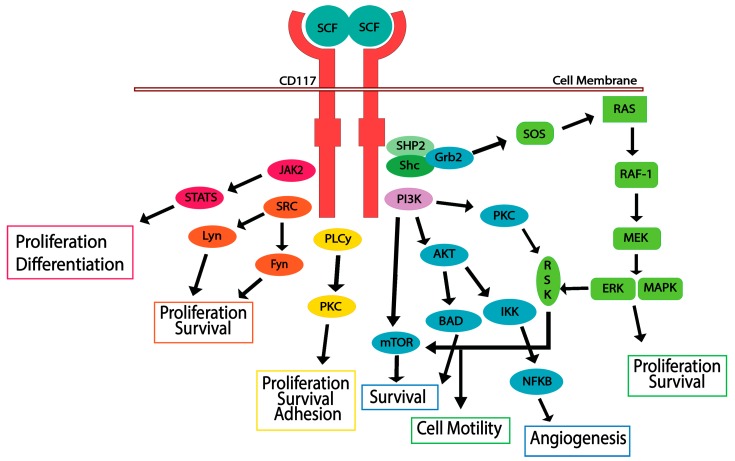
CD117 activation stimulates multiple signaling pathways. Stem cell factor (SCF) ligand binding to the CD117 receptor induces dimerization and downstream signaling, resulting in proliferation, differentiation, survival, adhesion, motility, and angiogenesis.

Activation of CD117 occurs when an SCF dimer binds to its extracellular domain. Inactive CD117 is found on the cell surface as a monomer; while SCF exists extracellularly as a dimer [40,74]. Upon the binding of SCF, the CD117 receptor forms a homodimer, causing autophosphorylation among specific tyrosine residues in the intracellular catalytic domain [24,75]. CD117 phosphorylation triggers several signal transduction pathways, including JAK/STAT, RAS/MAP kinase pathway, PI3 kinase, PLCγ pathway, and SRC pathway (Figure 2). Cell survival, proliferation, differentiation, and migration occur once CD117 is activated, requiring overlap of these pathways [22,30,42,59,76,77]. CD117 is then rapidly ubiquitinated by SOCS6 after autophosphorylation, resulting in internalization and degradation. The downstream pathways are discussed in detail below.

### 5.1. JAK/STAT Pathway

The JAK/STAT pathway plays a significant role in cell proliferation and differentiation in both murine and human cells. SCF binding induces rapid activation of JAK2 and stimulates the phosphorylation of STATs 1, 2, or 5. Once STATs are phosphorylated, they translocate to the nucleus, where they regulate transcription of target genes responsible for cell proliferation [78,79].

### 5.2. RAS/MAP Kinase Pathway

Activation of the RAS/MAP kinase cascade occurs when activated CD117 recruits adaptor proteins containing an SH-2 domain, such as GRB2, Shc, and SHP2. Grb2 will bind directly to CD117 at the phosphorylated Y703 and Y936 residues, or indirectly to Shc or SHP2 [26,80]. Once bound, the GRB2 will associate with SOS (Son-of-Sevenless), a guanine nucleotide exchange factor, and this complex activates the G-protein Ras [26,81]. Activation of Ras leads to the activation of Raf-1, which will activate MEK. MEK1/2 phosphorylates ERK1/2, which will phosphorylate and activate several transcription factors. The result of the activation of the RAS/MAP kinase cascade is regulation of cell proliferation, apoptosis, differentiation, adhesion, and mobility [22,82,83].

### 5.3. PI3-Kinase/Akt Pathway

PI3 kinase pathway is responsible for Akt and mTOR activity. This pathway is activated by directly interacting with CD117 at Tyr-721, or indirectly, by binding to the scaffold protein Gab2, which contacts the adapter protein Grb2 [30,84]. The PI3-K pathway is the primary pathway responsible for cell survival. Akt interacts with the pro-apoptotic factor BAD and causes inactivation, leading to cell survival. Further, CD117 phosphorylation and activation of the PI3 kinase and SRC pathways contributes to SCF-mediated cell motility [85].

### 5.4. SRC Family Kinase Pathways

The GNNK- splice variant of CD117 strongly activates SRC and the SRC family kinases (SFK). These kinases can interact with several tyrosine residues on CD117, but only Tyr568 is required for activation. SCF can also activate SFK; specifically, Lyn, Fyn, and PLCγ. Lyn activation increases the activity of cyclin dependent kinase 2 (CDK2) as well as phosphorylation of Rb, to promote cell proliferation [86,87]. While Lyn promotes cell proliferation, it was also demonstrated that Lyn can negatively regulate PI3-kinase/AKT pathway, although the underlying mechanism is still unknown [88]. While Lyn can negatively regulate the PI3-kinase pathway, Fyn can phosphorylate Akt downstream. Fyn also plays a role in activating PLCγ when interacting with the truncated form of CD117 (tr-KIT) through mouse oocyte activation [88,89].

### 5.5. PLCγ Pathway

Several studies show different docking sites for PLCγ. PLCγ can associate with the phosphorylated Tyr728, Tyr730, Tyr936, and Tyr900 residues of CD117 [90,91,92]. PIP2 is hydrolyzed by PLCγ to generate DAG and IP3. DAG activates PKC by binding, while IP3 causes the release of Ca^2+^. PKC has a role in cell survival, proliferation, and adhesion [30,93]. Thus, activation of the SCF/CD117 signaling axis can drive cell survival, proliferation, and motility; essential steps in cancer progression.

## 6. CD117 Regulation of Cancer Progression

**Figure 3:**
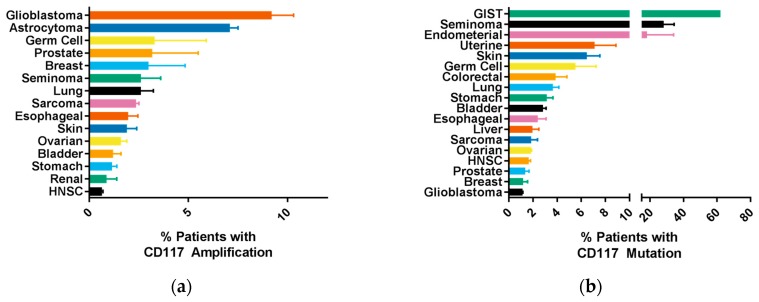
CD117 is amplified or mutated in a variety of cancers. Genomic datasets in cBioPortal [96,97] were examined for amplifications (**a**) or mutations (**b**) of CD117 (*KIT* gene). The mean percentage of patients with each cancer type with amplifications or mutations ± SEM are shown.

Overactivation of CD117 is the primary mutation seen in several cancer types, such as gastrointestinal tumors (GIST), mastocytosis, acute myelogenous leukemia (AML), and melanoma [24,30,40]. Recent studies and clinical trials suggested that CD117 can be used effectively for prognosis, particularly for predicting cancer metastasis and response to chemotherapy. Biomarkers involving CD117 were identified and studied across various tumor cell types [94,95]. In a single study, CD117 was expressed in 21% of breast cancers, 17% of colorectal cancers, 35% of sarcomas, 36% of renal cell carcinomas, 17% ovarian cancers, and 17% of hepatocellular tumors. While insignificant, there was a trend towards worse prognosis in these patients [95]. Furthermore, 63% of AML patients had CD117 mutations, while 89–100% of GIST patient expressed CD117 [40]. Figure 3 shows CD117 (*KIT* gene) amplification and mutation in several cancers using datasets available through cBioPortal [96,97]. Complete amplification, mutation, deletion, and alterations for the CD117 (*KIT* gene) and the SCF (*KITLG* gene) are available in Tables S1 and S2, respectively. Genetic variants of CD117 (as a result of exon deletions) identified poor prognosis in GIST patients following primary tumor resection [98,99,100]. A 2012 study of resected tumors from thirty-eight patients prior to treatment with imatinib found that 63% of tumors had mutations located on CD117 [101]. In concert, a 2017 study found that CD117 was expressed in 88% of surveyed cases where GIST had metastasized to bone, with the most common mutations in exon 11 and 13 [102]. These activating mutations, particularly in exon 11, were confirmed in similar studies analyzing GIST patients [103,104].

Beyond GIST, in patients with primary ovarian high-grade serous carcinoma, high expression of CD117 suggested shorter disease-free survival and peritoneal metastasis [105]. This accelerated progression resulted from the tumorigenic and chemoresistant nature of ovarian cancer cells with CD117-expressing phenotypes [106,107]. Recent studies found that CD117 positive cells in the circulation are predictive of advanced prostate cancer, with a positive correlation between CD117 expression and Gleason scores [14,108]. A 2008 study suggested a trend of increased expression of CD117 during prostate cancer metastasis to the bone; a follow-up study in 2015 by the same lab found a novel pathway linking CD117 expression with BRCA2 downregulation that induced bone metastasis of prostate cancer [16,109,110]. Co-expression of CD117 and associated stem cell factors and ligands in breast carcinomas and small cell lung cancers also play a role in autocrine growth and tumor cell proliferation [111,112]. Activating mutations and overexpression of the proto-oncogene CD117 are, therefore, essential factors in considering tumor growth and metastasis in multiple solid tumors that develop outside the bone microenvironment.

These findings are not consistent across all cancers, and the expression of CD117 may impact myeloid/erythroid-derived cancers differently than it does solid tumors. For example, CD117 expression has the opposite effect in multiple myelomas, which originate in the bone marrow. CD117 positive malignant plasma cells are linked to improved prognosis in patients with multiple myeloma [113,114,115]. This suggests a more complicated relationship between CD117 expression and cancer prognosis than initially suspected. In short, while the prognostic value of CD117 appears promising, it remains an area in need of additional study [116].

Complementing the role of CD117, SCF may also play a role in cancer progression. Particularly high levels of SCF are found in the bone marrow, one location for metastasis and thus, an SCF gradient may be one driver of bone metastasis. Bone marrow stromal cells and prostate cancer cells express both membrane and soluble SCF; however, BMSCs secrete much higher levels of the soluble SCF. Once exposed to bone marrow, which is high in SCF, PC3 prostate cancer cells started to express CD117 [16], indicating that the bone microenvironment might induce CD117 expression, leading to overexpression and metastasis. SCF production by hypoxic tissues induces CD117 positive myeloid cell mobilization, as well as homing [117]. Thus, an interplay between SCF and CD117 may drive cancer progression and metastasis.

## 7. CD117 Regulation of Cancer Cell “Stemness”

Studies suggest that CD117 plays an important role in cell differentiation and survival, particularly in its impact on CSCs. In a study on non-small cell lung cancer patients, tumor cells positively expressing CD117 exhibited CSC characteristics, such as self-renewal and chemoresistance [118]. Similar characteristics are seen in CD117 positive ovarian tumor cells in which CD117 expression is related to the “stemness” of particular cancer cells [107,119]. Beyond cancer, healthy and developing T-cells and B-cells gradually lose expression of CD117 as they differentiate and mature (thereby losing their “stemness”), further suggesting that CD117 signaling is needed to keep cell plasticity [22,120,121].

Activation of CD117 in cancer leads to the activation of many downstream signaling pathways, such as RAS/ERK, PI3-kinase, SRC, JAK/STAT, WNT, and NOTCH, and activation of these pathways are known to induce “stemness” or a stem-like phenotype. For example, activated tyrosine kinase SRC interacts with motifs on Akt-mTOR in acute myeloid leukemia (AML) cells, a process which upregulates signaling and stemness in AML [122,123,124]. In 2010, a study of human colon carcinoma and synovial sarcoma cell lines found that Ras/ERK pathways contributed in part to both the maintenance and acquisition of stemness in tumors [125]. The associations of CD117 (*KIT* gene) mutations with mutated signaling pathways genes are shown in Table S3 for prostate cancer as an example. As such, cells exhibiting “stemness” are those that share some, or all, properties of stem cells [126,127]. In fact, CD117 positive prostate cancer cells may be CSCs that express potential CSC markers Sox2 and Oct4. The cells can also generate tumors in serial tumor initiation experiments, a requirement for the classification as a CSC [15]. This ability to control “stemness” indicates that CD117 may be a marker for CSCs.

## 8. CD117 Resistance to Tyrosine Kinase Inhibitors

Tyrosine kinase inhibitors (TKIs) are being tested in a variety of cancers expressing CD117 and other related tyrosine kinase receptors. Many of these inhibitors were originally developed for other members of the type III tyrosine kinase receptor family. However, due to overlaps in receptor structure, many TKIs have specificity for CD117 as well (Table 1). In particular, the TKI imatinib (Gleevec) is a standard treatment that has demonstrated specificity for inhibiting CD117, among other tyrosine kinases, such as BCR-ABL [128,129]. Early studies on imatinib in vitro and in human patients with GIST confirmed the role of CD117 in cancer metastasis. In these studies, imatinib was well tolerated and effective at targeting the tyrosine kinase domain of CD117 [130,131,132]. Imatinib’s inhibitory effects on CD117 (coupled with its inhibition of indoleamine 2,3-dioxygenase, an immunosuppressive enzyme) have made it a first-line chemotherapeutic agent [133,134,135]. However, developing resistance to imatinib is not uncommon [136]. Unresectable metastatic imatinib-resistant GISTs led to the development of related TKIs such as sunitinib and regorafenib [137,138,139,140]. CD117 mutations in GIST are responsible for resistance to TKI treatment. Fourteen percent of GIST patients are initially resistant to imatinib, and 50% develop resistance within two years of therapy. For most patients, sunitinib will then be used and effective, unless these patients possess the D816H/V mutation, in which case they will be resistant to both TKIs. Imatinib works better on inactive CD117 and prevents activation, but does not bind to activated CD117 [141]. Failure of imatinib in the treatment of chronic myeloid leukemia (CML), which primarily inhibits BCR-ABL in this cancer cell line, led to the development of nilotinib as a second-line treatment, a drug that also exhibits anti-CD117 properties [142,143,144].

Clinical trials of imatinib and related TKIs are ongoing, with researchers also studying the effects on various cancer cell lines. Phase 3 randomized trials found that nilotinib was unsuccessful as either first-line therapy for GIST or as second-line therapy for imatinib-resistant GIST, relegating its use mainly to CML [165,166]. In clinical trials of patients with AIDS-associated Kaposi’s sarcoma, imatinib has demonstrated clinical benefit through its inhibition of both CD117 and platelet-derived growth factor (PDGF) [167,168,169]. Imatinib has also been shown to effectively treat melanoma that possesses an amplified or mutated CD117 oncogene [170,171]. The anti-angiogenesis properties of TKIs, such as imatinib, sunitinib, and pazopanib (all of which also target CD117), have been posited as promising therapies for epithelial ovarian cancer, with clinical trials demonstrating efficacy and tolerability in all three drugs [172,173]. To date, TKIs remain a focus of study, with both pilot and large-scale clinical trials reporting data on their potential benefits in metastatic melanoma, fibromatosis, and neuroendocrine tumors [174,175,176,177,178].

## 9. The Future of the SCF/CD117 Signaling Axis in Cancer Treatment

While there is continued study of the early generations of TKIs, despite their broad reactivity and off-target effects, research continues to develop inhibitors specific for each individual kinase expressed in cancer cells. Ongoing studies into TKI treatment efficacy require new tools for studying their effects in vivo and in vitro. Prior to phase 1 clinical trials, most treatments are tested in animal models and on human cell lines. Newer patient-derived xenografts are allowing for testing in primary human samples, while metastasis-on-chip models [179,180] permit high throughput screening of candidate compounds. The ability to directly target SCF-secreting or CD117-expressing cells may improve patient treatment, as CD117 activation and signaling are upregulated in a variety of tumors. Continued examination is also needed to define which tumor types and subset of patients would benefit from CD117 inhibition. A better understanding of the role of CD117 in cancer progression is necessary for determining which patients should be treated, and how the receptor can be targeted.

Moreover, information on CD117’s role in cancer progression would validate its use as a potential biomarker of CSCs in tissues and CTCs in the bloodstream. While multiple studies describe CD117-expressing cells as potential CTCs or metastatic tumor-initiating cells, the inability to accurately isolate CTCs has prevented characterization of these two populations. Tracking CD117-expressing cells in a liquid biopsy would allow for definitive data confirming CD117 as a CTC marker in a variety of cancers and provide a way to evaluate patients in future CD117 inhibitor testing. Multiple labs have been developing microfluidic chips to isolate and quantify CTCs based on cell size, electromagnetic changes, or cell surface marker expression [181,182], which could be used for CD117. The ability to enumerate CD117-expressing cells in tumors and circulation could lead to improved tracking of response to treatment and therapeutic resistance in patients treated with TKIs. More recently, inhibitors specifically targeting CD117 were developed and tested in vitro in preventing cancer cell proliferation and migration [15,183]. Further studies are needed to examine the effects of CD117 targeting in vivo and in phase 1 clinical trials. Furthermore, combinatory targeting of CD117 with its downstream pathways may have improved efficacy. By targeting the CD117-expressing CSC population, in combination with conventional treatments working on the non-CSC population, a greater proportion of the bulk tumor could be eradicated.

## Figures and Tables

**Table 1:** Specificity of tyrosine kinase inhibitors for CD117.

Drug Name	Trade Name	Select Targets (Other than CD117)	Bioavailability	Specificity for CD117	References
Imatinib	Gleevec/Glivec, STI571	BCR-Abl, RET, PDGF-R	98%	0.1 μM	[145,146,147]
Sunitinib	Sutent, SU11248	JAK/STAT, PDGF-R, Ras/MAPK, VEGFR	50% (fasting)	26 nM	[145,147,148,149]
Nilotinib	Tasigna	BCR-Abl, Lck	30%	N.A.	[145,150]
Dasatinib	Sprycel	BCR-Abl, Src	14–34%	13 nM	[145,147,151,152]
Axitinib	Inlyta	BCR-Abl, PDGFR, VEGFR	58%	1.7 nM	[145,153,154]
Masitinib	Masivet, Kinavet	FGFR, PDGFR	60% (animals)	200 ± 40 nM	[155,156,157]
Pazopanib	Votrient	FGFR, PDGFR, VEGFR	14–39%	146 nM	[145,147,158,159]
Toceranib	Palladia	PDGFR, VEGFR	77%	<10 nM	[160,161]
Cabozantinib	XL184	VEGFR, c-Met	74–93%	4.6 nM	[162]
Flumatinib	HH-GV-678	c-Abl, PDGFR	N.A.	2.66 μM	[163,164]

N.A. indicates not available.

## Supplementary Materials

Supplementary materials can be found at http://www.mdpi.com/2227-9059/6/1/31/s1.
