# Supplementary material for: CD117/c-kit in Cancer Stem Cell-Mediated Progression and Therapeutic Resistance
Source: Biomedicines. 2018 Mar 8;6(1):31. doi: 10.3390/biomedicines6010031 (PMC5874688; doi:10.3390/biomedicines6010031)
Supplement: Supplementary file 1 [file biomedicines-06-00031-s001.pdf]

Gene	Cancer Type	% amplification or mutation or deletion	# of Patients altered of total # of patients	Source data
KIT	Seminoma	34.4% Mutation<br>1.6% Amplification	23 of 64 patients	TCGA, Provisional
KIT	Mixed Germ Cell Tumor	5.9% Mutation<br>5.9% Amplification	1 of 17 patients	TCGA Provisional
KIT	Non-Seminomatous Germ Cell Tumor	2.1 % Mutation	1 of 48 patients	TCGA Provisional
KIT	Prostate Adenocarcinoma	17% amplification	18 of 109 patients	Broad/Cornell, Nat Genet 2012
KIT	Glioblastoma Multiforme	11% Amplification<br>1.1% Mutation	11 of 91 patients	TCGA, Nature 2008
KIT	Seminoma	3.6% Amplification<br>21.8% Mutation		MSK, JCO 2016
KIT	Non-Seminomatous Germ Cell Tumor	4% Mutation	5 of 125 cases	MSK, JCO 2016
KIT	Prostate Neuroendocrine Carcinoma	2.3% Mutation<br>11.4% Alteration	13.6% of 44 cases	Trento / Cornell / Broad 2016
KIT	Prostate Cancer	9.5% Alteration	9.5% of 63 cases	Trento / Cornell / Broad 2016
KIT	Glioma	10.5% Amplification<br>1.2% Mutation	29 of 248 cases	TCGA , Cell 2013
KIT	Undefined	3% Amplification	1 of 33 cases	TCGA , Cell 2013
KIT	Cutaneous Squamous Cell Carcinoma	10.3% Mutation	3 of 29 cases	DFIC, Clin Cancer Res 2015
KIT	Invasive Breast Carcinoma	10.3% Amplification	3 of 29 cases	British Columbia, Nature 2014
KIT	Glioblastoma Multiforme	9.2% Amplification<br>1.1% Mutation	28 of 273 cases	TCGA, Provisional
KIT	Desmoplastic Melanoma	10% Mutation	2 of 20 cases	Broad Institute, Nat Genet 2015
KIT	Uterine Carcinosarcoma /Uterine Malignant Mixed Mullerian Tumor	9.1% Mutation	2 of 22 cases	John Hopkins University, Nat Commun 2014
KIT	Lung Squamous Cell Carcinoma	5.1% Amplification<br>2.3% Mutation	15 of 177 cases	TCGA, Provisional

KIT	Lung Squamous Cell Carcinoma	4.5% Amplification<br>3.4% Mutation	15 of 178 cases	TCGA, Nature 2012
KIT	Melanoma	8.3% Mutation	2 out of 24 cases	Melanoma (Broad/Dana Farber, Nature 2012)
KIT	Uterine Endometrioid Carcinoma	5% Amplification<br>8.8% Muataion	18 out of 193 cases	Uterine Corpus Endometrial Carcinoma (TCGA, Nature 2013)
KIT	Non-Small Cell Lung Cancer	7.3% Mutation	3 of 41 cases	Genetic Characterization of NSCLC young adult patients (University of Turin, Lung Cancer 2016)
KIT	Mixed Cancer Types	4.2% Mutation<br>1.9% Amplification<br>1% Deletion	64 of 881 cases	Cancer Cell Line Encyclopedia (Novartis/Broad, Nature 2012)
KIT	Sezary Syndrome	11.5% Mutation	3 out of 26 cases	Cutaneous T Cell Lymphoma (Columbia U, Nat Genet 2015)
KIT	Uterine Endometrioid Carcinoma	8.7% Mutation	17 of 195 cases	Uterine Corpus Endometrial Carcinoma (TCGA, Provisional)
KIT	Salivary Gland Cancer	12% Amplification	6 of 50 cases	Recurrent and Metastatic Head & Neck Cancer (JAMA Oncology, 2016)
KIT	Skin Cancer, Non-Melanoma	10.5% Mutation	2 of 19 cases	Recurrent and Metastatic Head & Neck Cancer (JAMA Oncology, 2016)

KIT	Head and Neck Cancer	1.6% Mutation	1 of 62 cases	Recurrent and Metastatic Head & Neck Cancer (JAMA Oncology, 2016)
KIT	Malignant Peripheral Nerve Sheath Tumor	6.7% Amplification	1 of 15 cases	Malignant Peripheral Nerve Sheath Tumor (MSKCC, Nat Genet 2014)
KIT	Cutaneous Melanoma	3.1% Mutation<br>2.4% Amplification	19 of 287 cases	Skin Cutaneous Melanoma (TCGA, Provisional)
KIT	Small Cell Lung Cancer	6.4% Mutation	7 of 110 cases	Small Cell Lung Cancer (U Cologne, Nature 2015)
KIT	Diffuse Glioma	5% Amplification<br>0.8% Mutation	47 of 794 cases	Merged Cohort of LGG and GBM (TCGA, Cell 2016)
KIT	Cancer Of Unknown Primary	10% Mutation	3 of 30 cases	NCI-60 Cell Lines (NCI, Cancer Res. 2012)
KIT	Lung Squamous Cell Carcinoma	4.3% Amplification<br>3.5% Mutation<br>0.2% Deletion	41 of 484 cases	Pan-Lung Cancer (TCGA, Nat Genet 2016)
KIT	Lung Adenocarcinoma	1.4% Amplification<br>2% Mutation	23 of 660 cases	Pan-Lung Cancer (TCGA, Nat Genet 2016)

KIT	Colorectal Adenocarcinoma	5.6% Mutation	4 of 72 cases	Colorectal Adenocarcinoma (Genentech, Nature 2012)
KIT	Esophageal Adenocarcinoma	3.4% Mutation<br>2.3% Amplification	5 of 88 cases	Esophageal Carcinoma (TCGA, Provisional)
KIT	Esophageal Squamous Cell Carcinoma	3.1% Mutation<br>1% Deletion<br>1% Amplification	5 of 96 cases	Esophageal Carcinoma (TCGA, Provisional)
KIT	Sarcoma	2.9% Mutation<br>2.4% Amplification	11 of 207 cases	Sarcoma (MSKCC/Broad, Nat Genet 2010)
KIT	Cutaneous Melanoma	4.5% Mutation	3 of 66 cases	PTEN loss of function alterations are associated with intrinsic resistance to BRAF inhibitors in metastatic melanoma (JCO Precision Oncology,2017)
KIT	Stomach Adenocarcinoma	4.5% Mutation	1 of 22 cases	Stomach Adenocarcinoma (UHK, Nat Genet 2011)
KIT	Stomach Adenocarcinoma	3.1% Mutation<br>0.7% Deletion<br>0.3% Amplification	13 of 287 cases	Stomach Adenocarcinoma (TCGA, Nature 2014)

KIT	Esophagogastric Carcinoma	3.9% Mutation<br>2.6% Amplification	5 of 77 cases	TCGA data for Esophagus-Stomach Cancers (TCGA, Nature 2017)
KIT	Stomach Adenocarcinoma	2.1% Mutation<br>1.1% Amplification<br>0.5% Deletion	7 of 188 cases	TCGA data for Esophagus-Stomach Cancers (TCGA, Nature 2017)
KIT	Ovarian Cancer	1.9% Mutation<br>1.9% Amplification<br>0.3% Deletion	13 of 310 cases	Ovarian Serous Cystadenocarcinoma (TCGA, Provisional)
KIT	Cutaneous Melanoma	4.4% Mutation	4 of 91 cases	Skin Cutaneous Melanoma (Yale, Nat Genet 2012)
KIT	Acinar Cell Carcinoma of the Pancreas	4.3% Mutation	1 of 23 cases	Acinar Cell Carcinoma of the Pancreas (Johns Hopkins, J Pathol 2014)
KIT	Nerve Sheath Tumor	12.5% Mutation	1 of 8 cases	Sarcoma (TCGA, Provisional)
KIT	Soft Tissue Sarcoma	1.3% Mutation<br>2.6% Amplification	9 of 235 cases	Sarcoma (TCGA, Provisional)
KIT	Signet Ring Cell Carcinoma of the Stomach	22.2% Deletion	2 of 9 cases	Stomach Adenocarcinoma (TCGA,

KIT	Mucinous Stomach Adenocarcinoma	5% Mutation	1 of 20 cases	Stomach Adenocarcinoma (TCGA,
KIT	Tubular Stomach Adenocarcinoma	4.9% Mutation	3 of 61 cases	Stomach Adenocarcinoma (TCGA,
KIT	Diffuse Type Stomach Adenocarcinoma	1.4% Mutation<br>1.4% Amplification	3 of 70 cases	Stomach Adenocarcinoma (TCGA,
KIT	Stomach Adenocarcinoma	2.2% Mutation<br>0.9% Amplification	7 of 227 cases	Stomach Adenocarcinoma (TCGA,
KIT	Bladder Urothelial Carcinoma	2.4% Mutation<br>1.6% Amplification	5 of 127 cases	Bladder Urothelial Carcinoma (TCGA, Provisional)
KIT	Lung Adenocarcinoma	2.2% Mutation<br>1.7% Amplification	9 of 230 cases	Lung Adenocarcinoma (TCGA, Provisional)
KIT	Metastatic Breast Cancer	1.9% Mutation<br>1.9% Amplification	8 of 213 cases	Mutational profiles of metastatic breast cancer (France, 2016)
KIT	Acute Myeloid Leukemia	3.7% Mutation	7 of 188 cases	Acute Myeloid Leukemia (TCGA, Provisional)

KIT	Pancreatic Adenocarcinoma	0.9% Amplification<br>2.8% Deletion	4 of 109 cases	Pancreatic Cancer (UTSW, Nat Commun 2015)
KIT	Acute Myeloid Leukemia	3.7% Mutation	7 of 191 cases	Acute Myeloid Leukemia (TCGA, NEJM 2013)
KIT	Prostate Adenocarcinoma	1.8% Mutation<br>1.8% Amplification	2 of 56 cases	Prostate Adenocarcinoma (Broad/Cornell, Cell 2013)
KIT	Colorectal Adenocarcinoma	3.6% Mutation	22 of 619 cases	Colorectal Adenocarcinoma (DFCI, Cell Reports 2016)
KIT	Lung Adenocarcinoma (Non-Small Cell Lung Cancer)	2.2% Mutation<br>1.3% Amplification	8 of 223 cases	Lung Adenocarcinoma (TCGA, Nature 2014)
KIT	Small Cell Lung Cancer	3.5% Mutation	1 of 29 cases	Small Cell Lung Cancer (CLCGP, Nat Genet 2012)
KIT	Gastrointestinal Stromal Tumor	62% Mutation<br>0.7% Amplification<br>0.7% Deletion	91 of 137 cases	MSK-IMPACT Clinical Sequencing Cohort (MSKCC, Nat Med 2017)
KIT	Penile Cancer	14.3% Mutation	1 of 7 cases	MSK-IMPACT Clinical Sequencing Cohort (MSKCC, Nat Med 2017)

KIT	Germ Cell Tumor	10.1% Mutation<br>0.7% Amplification	33 of 288 cases	MSK-IMPACT Clinical Sequencing Cohort (MSKCC, Nat Med 2017)
KIT	undefined	3.5% Mutation<br>1.8% Deletion	5 of 57 cases	MSK-IMPACT Clinical Sequencing Cohort (MSKCC, Nat Med 2017)
KIT	Glioma	1.3% Mutation<br>6.1% Amplification	42 of 553 cases	MSK-IMPACT Clinical Sequencing Cohort (MSKCC, Nat Med 2017)
KIT	Melanoma	3.7% Mutation<br>1.4% Amplification	25 of 350 cases	MSK-IMPACT Clinical Sequencing Cohort (MSKCC, Nat Med 2017)
KIT	Small Cell Lung Cancer	4.4% Mutation<br>2.2% Amplification	6 of 91 cases	MSK-IMPACT Clinical Sequencing Cohort (MSKCC, Nat Med 2017)
KIT	Salivary Gland Cancer	6.1% Amplification	7 of 114 cases	MSK-IMPACT Clinical Sequencing Cohort (MSKCC, Nat Med 2017)
KIT	Skin Cancer, Non-Melanoma	6.1% Mutation	9 of 148 cases	MSK-IMPACT Clinical Sequencing Cohort (MSKCC, Nat Med 2017)
KIT	Thymic Tumor	5.6% Mutation	1 of 18 cases	MSK-IMPACT Clinical Sequencing Cohort (MSKCC, Nat Med 2017)

KIT	Adrenocortical Carcinoma	3.8% Mutation	1 of 26 cases	MSK-IMPACT Clinical Sequencing Cohort (MSKCC, Nat Med 2017)
KIT	Bladder Cancer	3.5% Mutation	15 of 423 cases	MSK-IMPACT Clinical Sequencing Cohort (MSKCC, Nat Med 2017)
KIT	Soft Tissue Sarcoma	1.4% Mutation<br>2.1% Amplification	15 of 434 cases	MSK-IMPACT Clinical Sequencing Cohort (MSKCC, Nat Med 2017)
KIT	Endometrial Cancer	3.2% Mutation	7 of 218 cases	MSK-IMPACT Clinical Sequencing Cohort (MSKCC, Nat Med 2017)
KIT	Anal Cancer	3.1% Mutation	1 of 32 cases	MSK-IMPACT Clinical Sequencing Cohort (MSKCC, Nat Med 2017)
KIT	Bone Cancer	3% Amplification	4 of 134 cases	MSK-IMPACT Clinical Sequencing Cohort (MSKCC, Nat Med 2017)
KIT	Colorectal Cancer	2.6% Mutation<br>0.1% Deletion	27 of 1006 cases	MSK-IMPACT Clinical Sequencing Cohort (MSKCC, Nat Med 2017)
KIT	Appendiceal Cancer	1.3% Mutation<br>1.3% Amplification	2 of 79 cases	MSK-IMPACT Clinical Sequencing Cohort (MSKCC, Nat Med 2017)

KIT	Non-Small Cell Lung Cancer	2% Mutation<br>0.4% Amplification	39 of 1659 cases	MSK-IMPACT Clinical Sequencing Cohort (MSKCC, Nat Med 2017)
KIT	Head and Neck Cancer	1.7% Mutation<br>0.6% Amplification	4 of 173 cases	MSK-IMPACT Clinical Sequencing Cohort (MSKCC, Nat Med 2017)
KIT	Ovarian Cancer	1.8% Mutation<br>0.4% Deletion	5 of 224 cases	MSK-IMPACT Clinical Sequencing Cohort (MSKCC, Nat Med 2017)
KIT	Cancer of Unknown Primary	1.6% Mutation	3 of 184 cases	MSK-IMPACT Clinical Sequencing Cohort (MSKCC, Nat Med 2017)
KIT	Esophagogastric Cancer	1.5% Mutation	5 of 341 cases	MSK-IMPACT Clinical Sequencing Cohort (MSKCC, Nat Med 2017)
KIT	Non-Hodgkin Lymphoma	1.2% Deletion	2 of 165 cases	MSK-IMPACT Clinical Sequencing Cohort (MSKCC, Nat Med 2017)
KIT	Hepatobiliary Cancer	1.2% Mutation	4 of 338 cases	MSK-IMPACT Clinical Sequencing Cohort (MSKCC, Nat Med 2017)
KIT	Adenoid Cystic Carcinoma	3.3% Amplification	2 of 60 cases	Adenoid Cystic Carcinoma (MSKCC, Nat Genet 2013)

KIT	Prostate Adenocarcinoma	2% Mutation<br>1.3% Amplification	5 of 150 cases	Metastatic Prostate Cancer, SU2C/PCF Dream Team (Robinson et al., Cell 2015)
KIT	Cutaneous Melanoma	3.3% Mutation	4 of 121 cases	Skin Cutaneous Melanoma (Broad, Cell 2012)
KIT	Lung Adenocarcinoma	3.3% Mutation	6 of 182 cases	Lung Adenocarcinoma (Broad, Cell 2012)
KIT	Anaplastic Oligoastrocytoma	2.2% Mutation<br>6.7% Amplification	4 of 45 cases	Brain Lower Grade Glioma (TCGA, Provisional)
KIT	Anaplastic Astrocytoma	7.5% Amplification	5 of 67 cases	Brain Lower Grade Glioma (TCGA, Provisional)
KIT	High-Grade Serous Ovarian Cancer	1.9% Mutation<br>1.3% Amplification	10 of 316 cases	Ovarian Serous Cystadenocarcinoma (TCGA, Nature 2011)
KIT	Bladder Urothelial Carcinoma	2.4% Mutation<br>0.8% Amplification	4 of 127 cases	Bladder Urothelial Carcinoma (TCGA, Nature 2014)
KIT	Bladder Urothelial Carcinoma	3% Mutation	1 of 33 cases	Bladder Cancer, Plasmacytoid Variant (MSKCC, Nat Genet 2016)

KIT	Hepatocellular Carcinoma	3.1% Mutation	11 of 355 cases	Liver Hepatocellular Carcinoma (TCGA, Provisional)
KIT	Intrahepatic Cholangiocarcinoma	3.4% Mutation	1 of 29 cases	Cholangiocarcinoma (TCGA, Provisional)
KIT	Colorectal Adenocarcinoma	2.8% Mutation	6 of 212 cases	Colorectal Adenocarcinoma (TCGA, Nature 2012)
KIT	Head and Neck Squamous Cell Carcinoma	2.2% Mutation<br>0.6% Amplification	14 of 504 cases	Head and Neck Squamous Cell Carcinoma (TCGA, Provisional)
KIT	Mucinous Adenocarcinoma of the Colon and Rectum	8.7% Mutation	2 of 23 cases	Colorectal Adenocarcinoma (TCGA, Provisional)
KIT	Colon Adenocarcinoma	2.3% Mutation	3 of 129 cases	Colorectal Adenocarcinoma (TCGA, Provisional)
KIT	Rectal Adenocarcinoma	1.5% Mutation	1 of 68 cases	Colorectal Adenocarcinoma (TCGA, Provisional)
KIT	Endometrioid Carcinoma	50% Mutation	1 of 2 cases	Cervical Squamous Cell Carcinoma and Endocervical Adenocarcinoma (TCGA, Provisional)

KIT	Endocervical Adenocarcinoma	3.8% Mutation	1 of 26 cases	Cervical Squamous Cell Carcinoma and Endocervical Adenocarcinoma (TCGA, Provisional)
KIT	Cervical Squamous Cell Carcinoma	0.6% Mutation<br>0.6% Amplification<br>0.6% Deletion	3 of 155 cases	Cervical Squamous Cell Carcinoma and Endocervical Adenocarcinoma (TCGA, Provisional)
KIT	Small Cell Lung Cancer	2.4% Mutation	1 of 42 cases	Small Cell Lung Cancer (Johns Hopkins, Nat Genet 2012)
KIT	Head and Neck Squamous Cell Carcinoma	1.4% Mutation<br>0.7% Amplification	6 of 279 cases	Head and Neck Squamous Cell Carcinoma (TCGA, Nature 2015
KIT	Stomach Adenocarcinoma	2% Mutation	2 of 100 cases	Stomach Adenocarcinoma (Pfizer and UHK, Nat Genet 2014)
KIT	Prostate Adenocarcinoma	1% Mutation<br>1% Amplification	2 0f 103 cases	Prostate Adenocarcinoma (MSKCC, Cancer Cell 2010)
KIT	Unclassified Renal Cell Carcinoma	1.9% Amplification	1 of 52 cases	Unclassified Renal Cell Carcinoma (MSKCC 2016)

KIT	Breast Invasive Carcinoma	1% Mutation<br>0.7% Amplification<br>0.1% Deletion	15 of 816 cases	Breast Invasive Carcinoma (TCGA, Cell 2015)
KIT	Summary for Uterine Carcinosarcoma /Uterine Malignant Mixed Mullerian Tumor	1.8% Mutation	1 of 56 cases	Uterine Carcinosarcoma (TCGA, Provisional)
KIT	Nasopharyngeal Carcinoma	1.8% Mutation	1 of 56 cases	Nasopharyngeal Carcinoma (Singapore, Nat Genet 2014)
KIT	Breast Cancer	0.7% Mutation<br>0.9% Amplification<br>0.1% Deletion	17 of 962 cases	Breast Invasive Carcinoma (TCGA, Provisional)
KIT	Diffuse Large B-Cell Lymphoma	1.7% Mutation	1 of 58 cases	Diffuse Large B-Cell Lymphoma (Broad, PNAS 2012)
KIT	Prostate Adenocarcinoma	1.6% Multiple Alterations	1 of 61 cases	Prostate Adenocarcinoma, Metastatic (Michigan, Nature 2012)
KIT	Prostate Adenocarcinoma	0.7% Mutation<br>0.7% Amplification	2 of 136 cases	Prostate Adenocarcinoma (Fred Hutchinson CRC, Nat Med 2016)
KIT	Prostate Adenocarcinoma	0.4% Mutation<br>0.2% Amplification<br>0.8% Deletion	7 of 492 cases	Prostate Adenocarcinoma (TCGA, Provisional)

KIT	Esophagogastric Cancer	1.4% Mutation	2 of 145 cases	Esophageal Adenocarcinoma (Broad, Nat Genet 2013)
KIT	Head and Neck Squamous Cell Carcinoma	1.4% Mutation	1 of 74 cases	Head and Neck Squamous Cell Carcinoma (Broad, Science 2011)
KIT	Hepatocellular Carcinoma	0.9% Mutation<br>0.4% Amplification	3 of 231 cases	Liver Hepatocellular Carcinoma (AMC, Hepatology 2014)
KIT	Esophagogastric Adenocarcinoma	1.3% Mutation	1 of 78 cases	Gastric Adenocarcinoma (TMUCIH, PNAS 2015)
KIT	Melanoma	1.3% Deletion	1 of 79 cases	Uveal Melanoma (TCGA, Provisional)
KIT	Hepatocellular Carcinoma	1.2% Mutation	3 of 243 cases	Hepatocellular Carcinomas (Inserm, Nat Genet 2015)
KIT	Pheochromocytoma	0.7% Mutation<br>0.7% Amplification	2 of 135 cases	Pheochromocytoma and Paraganglioma (TCGA, Provisional)
KIT	Breast Cancer	1.2% Amplification<br>0% Deletion	25 of 2050 cases	Breast Cancer (METABRIC, Nature 2012 & Nat Commun 2016)

KIT	Prostate Adenocarcinoma	0.3% Amplification<br>0.9% Deletion	4 of 333 cases	Prostate Adenocarcinoma (TCGA, Cell 2015)
KIT	Renal Clear Cell Carcinoma	0.7% Mutation<br>0.5% Amplification	5 of 418 cases	Kidney Renal Clear Cell Carcinoma (TCGA, Nature 2013)
KIT	Pleural Mesothelioma, Epithelioid Type	1.8% Mutation	1 of 57 cases	Mesothelioma (TCGA, Provisional)
KIT	Esophageal Squamous Cell Carcinoma	1.1% Mutation	1 of 88 cases	Esophageal Squamous Cell Carcinoma (ICGC, Nature 2014)
KIT	Breast Invasive Carcinoma	1% Mutation	5 of 482 cases	Breast Invasive Carcinoma (TCGA, Nature 2012)
KIT	Pancreatic Neuroendocrine Tumor	1% Mutation	1 of 98 cases	Whole-Genome Sequencing of Pancreatic Neuroendocrine Tumors (Nature, 2017)
KIT	Bladder Urothelial Carcinoma	1% Mutation	1 of 99 cases	Bladder Urothelial Carcinoma (BGI, Nat Genet 2013)
KIT	Multiple Myeloma	1% Mutation	2 of 197 cases	Multiple Myeloma (Broad, Cancer Cell 2014)

KIT	Pediatric Ewing Sarcoma	1% Mutation	1 of 101 cases	Pediatric Ewing Sarcoma (DFCI, Cancer Discov 2014)
KIT	Clear Cell Renal Cell Carcinoma	0.9% Mutation	1 of 106 cases	Clear Cell Renal Cell Carcinoma (U Tokyo, Nat Genet 2013)
KIT	Bladder Cancer	0.9% Mutation	1 of 109 cases	Bladder Cancer (MSKCC, Eur Urol 2014)
KIT	Thymoma	0.8% Mutation	1 of 123 cases	Thymoma (TCGA, Provisional)
KIT	Pancreatic Adenocarcinoma	0.8% Mutation	3 of 383 cases	Pancreatic Adenocarcinoma (QCMG, Nature 2016)
KIT	Esophageal Squamous Cell Carcinoma	0.7% Mutation	1 of 137 cases	Esophageal Squamous Cell Carcinoma (UCLA, Nat Genet 2014)
KIT	Pancreatic Adenocarcinoma	0.7% Mutation	1 of 149 cases	Pancreatic Adenocarcinoma (TCGA, Provisional)

KIT	Chronic Lymphocytic Leukemia	0.6% Mutation	1 of 160 cases	Chronic Lymphocytic Leukemia (Broad, Cell 2013)
KIT	Lung Adenocarcinoma	0.6% Mutation	1 of 163 cases	Lung Adenocarcinoma (TSP, Nature 2008)
KIT	MSK-IMPACT Clinical Sequencing Cohort	0.4% Mutation	2 of 501 cases	MSK-IMPACT Clinical Sequencing Cohort (MSKCC): Prostate Cancer
KIT	Kidney Renal Clear Cell Carcinoma	0.2% Amplification	1 of 448 cases	Kidney Renal Clear Cell Carcinoma (TCGA, Provisional)
KIT	Genomic Hallmarks of Prostate Adenocarcinoma	0.2% Mutation	1 of 449 cases	Genomic Hallmarks of Prostate Adenocarcinoma (CPC-GENE, Nature 2017)
KIT	Chronic Lymphocytic Leukemia	0.2% Mutation	1 of 506 cases	Chronic Lymphocytic Leukemia (IUOPA, Nature 2015)

Table S1. Mutations and Amplifications of CD117/KIT in Cancer Datasets. Using cBioPortal the percentage of amplifications, mutations, deletions, and alterations were mined for the *KIT* gene across all available cancers. Summarized data is shown in the table.

Gene	Cancer Type	% amplification or mutation or deletion	# of Patients altered of total # of patients	Source data
KITLG	Prostate Cancer	14.3% Amplification<br>1.6% Mutation	10 of 63 patients	Neuroendocrine Prostate Cancer (Trento/Cornell/ Broad 2016)
KITLG	Prostate Neuroendocrine Carcinoma	13.6% Amplification	6 of 44 patients	Neuroendocrine Prostate Cancer (Trento/Cornell/ Broad 2016)
KITLG	Pancreatic Adenocarcinoma	0.9% Amplification<br>7.3% Deletion	9 of 109 patients	Pancreatic Cancer (UTSW, Nat Commun 2015)
KITLG	Prostate Cancer	50% Mutation	1 of 2 patients	NCI-60 Cell Lines (NCI, Cancer Res. 2012)
KITLG	Cancer of Unknown Primary	6.2% Mutation	3 of 30 patients	NCI-60 Cell Lines (NCI, Cancer Res. 2012)
KITLG	Mutational profiles of metastatic breast cancer	7% Amplification<br>0.5% Deletion	16 of 213 patients	Matic breast cancer (France, 2016)
KITLG	Invasive Breast Carcinoma	6.9% Amplification	2 of 29 patients	Breast cancer patient xenografts (British Columbia, Nature 2014)
KITLG	Sarcoma	6.3% Amplification	13 of 207 patients	Sarcoma (MSKCC/Broad,

				Nat Genet 2010)
KITLG	Soft Tissue Sarcoma	6% Amplification 0.4% Deletion	15 of 235 patients	Sarcoma (TCGA, Provisional)
KITLG	Prostate Adenocarcinoma	5.4% Amplification	3 of 56 patients	Prostate Adenocarcinoma (Broad/Cornell, Cell 2013)
KITLG	Acral Melanoma	5.3% Amplification	2 of 38 patients	Integrated genomic analyses reveal frequent TERT aberrations in acral melanoma. (Genome Res 2017)
KITLG	Prostate Adenocarcinoma	4.4% Amplification	6 of 136 patients	Prostate Adenocarcinoma (Fred Hutchinson CRC, Nat Med 2016)
KITLG	Cutaneous Melanoma	0.3% Amplification 3.1% Mutation	10 of 287 patients	Skin Cutaneous Melanoma (TCGA, Provisional)
KITLG	Diffuse Large B-Cell Lymphoma	3.4% Mutation	2 of 58 patients	Diffuse Large B-Cell Lymphoma (Broad, PNAS 2012)
KITLG	Adrenocortical Carcinoma	3.4% Amplification	3 of 87 patients	Adrenocortical Carcinoma (TCGA,

KITLG	Cutaneous Melanoma	3.3% Mutation	4 of 121 patients	Skin Cutaneous Melanoma (Broad, Cell 2012)
KITLG	Ovarian Cancer	1.9% Amplification<br>0.3% Mutation<br>0.3% Deletion	8 of 310 patients	Ovarian Serous Cystadenocarcinoma (TCGA, Provisional)
KITLG	Bladder Urothelial Carcinoma	1.6% Amplification<br>0.8% Mutation	3 of 127 patients	Bladder Urothelial Carcinoma (TCGA, Provisional)
KITLG	Bladder Urothelial Carcinoma	1.6% Amplification<br>0.8% Mutation	3 of 127 patients	Bladder Urothelial Carcinoma (TCGA, Nature 2014)
KITLG	Esophagogastric Cancer	0.5% Amplification<br>1.1% Mutation<br>0.5% Deletion	4 of 183 patients	Esophageal Carcinoma (TCGA, Provisional)
KITLG	Non-Hodgkin Lymphoma	2.1% Mutation	1 of 47 patients	Lymphoid Neoplasm Diffuse Large B-cell Lymphoma (TCGA, Provisional)
KITLG	Breast Invasive Carcinoma	1.5% Amplification<br>0.4% Mutation	16 of 816 patients	Breast Invasive Carcinoma (TCGA, Cell 2015)
KITLG	Prostate	1.9% Amplification	2 of 103 patients	Prostate

	Adenocarcinoma			Adenocarcinoma (MSKCC, Cancer Cell 2010)
KITLG	Uterine Carcinosarcoma/Uterine Malignant Mixed Mullerian Tumor	1.8% Mutation	1 of 56 patients	Uterine Carcinosarcoma (TCGA, Provisional)
KITLG	Breast Cancer	1.5% Amplification<br>0.3% Mutation	17 of 962 patients	Breast Invasive Carcinoma (TCGA, Provisional)
KITLG	Stomach Adenocarcinoma	1% Mutation<br>0.7% Deletion	5 of 287 patients	Stomach Adenocarcinoma (TCGA, Nature 2014)
KITLG	Adenoid Cystic Carcinoma	1.7% Amplification	1 of 60 patients	Adenoid Cystic Carcinoma (MSKCC, Nat Genet 2013)
KITLG	Uterine Serous Carcinoma/Uterine Papillary Serous Carcinoma	2.3% Amplification	1 of 43 patients	Uterine Corpus Endometrial Carcinoma (TCGA, Nature 2013)
KITLG	Uterine Endometrioid Carcinoma	1.3% Mutation	3 of 193 patients	Uterine Corpus Endometrial Carcinoma (TCGA, Nature 2013)
KITLG	Uterine Serous Carcinoma/Uterine Papillary Serous Carcinoma	2.3% Amplification	1 of 43 patients	Uterine Corpus Endometrial Carcinoma (TCGA, Provisional)

KITLG	Uterine Endometrioid Carcinoma	1.5% Mutation	3 of 195 patients	Uterine Corpus Endometrial Carcinoma (TCGA, Provisional)
KITLG	Prostate Adenocarcinoma	1.6% Amplification	1 of 61 patients	Prostate Adenocarcinoma, Metastatic (Michigan, Nature 2012)
KITLG	Mixed Cancer Types	1.2% Amplification 0.3% Deletion	14 of 881 patients	Cancer Cell Line Encyclopedia (Novartis/Broad, Nature 2012)
KITLG	Breast Invasive Carcinoma	1.5% Amplification	7 of 482 patients	Breast Invasive Carcinoma (TCGA, Nature 2012)
KITLG	Astrocytoma	6.5% Deletion	2 of 31 patients	Brain Lower Grade Glioma (TCGA, Provisional)
KITLG	Oligodendroglioma	1.5% Deletion	1 of 66 patients	Brain Lower Grade Glioma (TCGA, Provisional)
KITLG	Anaplastic Astrocytoma	1.5% Deletion	1 of 67 patients	Brain Lower Grade Glioma (TCGA, Provisional)
KITLG	Hepatobiliary Cancer	0.8% Amplification 0.5% Mutation	5 of 365 patients	Liver Hepatocellular Carcinoma (TCGA,

				Provisional)
KITLG	Breast Cancer	1.3% Amplification<br>0% Deletion	28 of 2050 patients	Breast Cancer (METABRIC, Nature 2012 & Nat Commun 2016)
KITLG	Mucinous Adenocarcinoma of the Colon and Rectum	4.3% Mutation	1 of 23 patients	Colorectal Adenocarcinoma (TCGA, Provisional)
KITLG	Colon Adenocarcinoma	0.8% Mutation<br>0.8% Deletion	2 of 129 patients	Colorectal Adenocarcinoma (TCGA, Provisional)
KITLG	Pancreatic Cancer	0.7% Amplification<br>0.7% Deletion	2 of 148 patients	Pancreatic Adenocarcinoma (TCGA, Provisional)
KITLG	Lung Adenocarcinoma	0.4% Mutation<br>0.9% Deletion	3 of 230 patients	Lung Adenocarcinoma (TCGA, Provisional)
KITLG	Non-Small Cell Lung Cancer	0.4% Mutation<br>0.9% Deletion	3 of 223 patients	Lung Adenocarcinoma (TCGA, Nature 2014)
KITLG	Mucinous Stomach Adenocarcinoma	5% Deletion	1 of 20 patients	Stomach Adenocarcinoma (TCGA, Provisional)
KITLG	Stomach Adenocarcinoma	1.8% Mutation	4 of 227 patients	Stomach Adenocarcinoma (TCGA,

				Provisional)
KITLG	High-Grade Serous Ovarian Cancer	0.6% Amplification<br>0.3% Mutation<br>0.3% Deletion	4 of 316 patients	Ovarian Serous Cystadenocarcinoma (TCGA, Nature 2011)
KITLG	Pleural Mesothelioma, Epithelioid Type	1.8% Amplification	1 of 57 patients	Mesothelioma (TCGA, Provisional)
KITLG	Cutaneous Melanoma	1.1% Mutation	1 of 91 patients	Skin Cutaneous Melanoma (Yale, Nat Genet 2012)
KITLG	Glioblastoma Multiforme	1.1% Mutation	1 of 91 patients	Glioblastoma (TCGA, Nature 2008)
KITLG	Cervical Squamous Cell Carcinoma	0.6% Mutation<br>0.6% Deletion	2 of 155 patients	Cervical Squamous Cell Carcinoma and Endocervical Adenocarcinoma (TCGA, Provisional)
KITLG	Prostate Adenocarcinoma	1% Amplification	5 of 492 patients	Prostate Adenocarcinoma (TCGA, Provisional)
KITLG	Bladder Urothelial Carcinoma	1% Mutation	1 of 99 patients	Bladder Urothelial Carcinoma (BGI, Nat Genet 2013)
KITLG	Stomach Adenocarcinoma	1% Mutation	1 of 100 patients	Stomach Adenocarcinoma (Pfizer and UHK, Nat Genet 2014)

KITLG	Colorectal Adenocarcinoma	0.9% Amplification	2 of 212 patients	Colorectal Adenocarcinoma (TCGA, Nature 2012)
KITLG	Lung Adenocarcinoma	0.2% Amplification<br>1.1% Mutation<br>0.3% Deletion	10 of 660 patients	Pan-Lung Cancer (TCGA, Nat Genet 2016)
KITLG	Esophagogastric Carcinoma	1.3% Deletion	1 of 77 patients	TCGA data for Esophagus-Stomach Cancers (TCGA, Nature 2017)
KITLG	Kidney Renal Clear Cell Carcinoma	0.4% Mutation<br>0.2% Deletion	3 of 448 patients	Kidney Renal Clear Cell Carcinoma (TCGA, Provisional)
KITLG	Metastatic Prostate Cancer	0.7% Amplification	1 of 150 patients	Metastatic Prostate Cancer, SU2C/PCF Dream Team (Robinson et al., Cell 2015)
KITLG	Ampullary Carcinoma	1.6% Mutation	1 of 62 patients	Ampullary Carcinoma (Baylor College of Medicine, Cell Reports 2016)
KITLG	Lung Adenocarcinoma	0.5% Mutation	1 of 182 patients	Lung Adenocarcinoma (Broad, Cell 2012)
KITLG	Acute Myeloid Leukemia	0.5% Deletion	1 of 188 patients	Acute Myeloid Leukemia

				(TCGA, Provisional)
KITLG	Acute Myeloid Leukemia	0.5% Deletion	1 of 191 patients	Acute Myeloid Leukemia (TCGA, NEJM 2013)
KITLG	Kidney Renal Clear Cell Carcinoma	0.5% Mutation	2 of 418 patients	Kidney Renal Clear Cell Carcinoma (TCGA, Nature 2013)
KITLG	Liver Hepatocellular Carcinoma	0.4% Mutation	1 of 231 patients	Liver Hepatocellular Carcinoma (AMC, Hepatology 2014)
KITLG	Hepatocellular Carcinomas	0.4% Mutation	1 of 243 patients	Hepatocellular Carcinomas (Inserm, Nat Genet 2015)
KITLG	Head and Neck Squamous Cell Carcinoma	0.2% Amplification 0.2% Mutation	2 of 504 patients	Head and Neck Squamous Cell Carcinoma (TCGA, Provisional)
KITLG	Glioblastoma Multiforme	0.4% Deletion	1 of 273 patients	Glioblastoma Multiforme (TCGA, Provisional)
KITLG	Head and Neck Squamous Cell Carcinoma	0.4% Amplification	1 of 279 patients	Head and Neck Squamous Cell Carcinoma (TCGA, Nature 2015)

KITLG	Glioblastoma	0.4% Deletion	1 of 281 patients	Glioblastoma (TCGA, Cell 2013)
KITLG	Merged Cohort of LGG and GBM	0.3% Deletion	2 of 794 patients	Merged Cohort of LGG and GBM (TCGA, Cell 2016)
KITLG	Papillary Thyroid Carcinoma	0.3% Amplification	1 of 399 patients	Papillary Thyroid Carcinoma (TCGA, Cell 2014)
KITLG	Colorectal Adenocarcinoma	0.2% Mutation	1 of 619 patients	Colorectal Adenocarcinoma (DFCI, Cell Reports 2016)

Table S2. Mutations and Amplifications of SCF/KITLG in Cancer Datasets. Using cBioPortal the percentage of amplifications, mutations, deletions, and alterations were mined for the *KITLG* gene across all available cancers. Summarized data is shown in the table.

Genes	Cancer Type	% amplification or mutation or deletion	# of Patients altered of total # of patients	Co-occurrence	Source data
KIT AKT1	Neuroendocrine Prostate Cancer	31% amplification on KIT+AKT1	24 of 77 patients	Significant tendency p=0.016	Trento/Cornell/ Broad 2016
KIT AKT1	Prostate adenocarcinoma	0.4% mutation on KIT+AKT1	2 of 449 patients	Tendency towards mutual exclusivity p= 0.998	CPC-GENE, Nature 2017
KIT AKT1	Prostate adenocarcinoma	1.6% mutation on KIT+AKT1	7 of 451 patients	Tendency towards mutual exclusivity p= 0.980	MSK, JCO Precision Oncology 2017
KIT AKT1	Prostate adenocarcinoma	7% mutation and amplification on KIT+AKT1	10 of 150 patients	Tendency towards mutual exclusivity p=0.842	Robinson et al, Cell 2015
KIT AKT1	Prostate adenocarcinoma	4% mutation and amplification on KIT only	2 of 56 patients	Tendency towards co-occurrence p=1.000	Broad/Cornell, Cell 2013
KIT AKT1	Prostate adenocarcinoma	17% mutation and amplification on KIT+AKT1	19 0f 109 patients	Tendency towards mutual exclusivity p=0.835	Broad/Cornell, Nat Genet 2012
KIT AKT1	Prostate adenocarcinoma	7% mutation and amplification on KIT+AKT1	4 of 54 patients	Tendency towards co-occurrence p=0.072	Fred Hutchinson CRC, Nat Med 2016
KIT AKT1	Prostate adenocarcinoma	1.9% mutation and amplification on KIT only	2 of 103 patients	Tendency towards co-occurrence p=1.000	MSKCC, Cancer Cell 2010

KIT AKT1	Prostate adenocarcinoma	3% mutation, amplification and deletion on KIT+AKT1	11 of 333 patients	Tendency towards mutual exclusivity p=0.918	TCGA, Cell 2015
KIT AKT1	Prostate adenocarcinoma	2.8% mutation, amplification and deletion on KIT+AKT1	14 of 492 patients	Tendency toward mutual exclusivity p=0.904	TCGA, Provisional
KIT AKT1	Prostate adenocarcinoma	0% alterations	0 of 104 patients	Tendency towards co-occurrence p=1.000	MSKCC, PNAS 2014
KIT AKT1	Prostate adenocarcinoma	0% alterations	0 of 7 patients	Tendency towards co-occurrence p=1.000	MSKCC, Cell 2014
KIT AKT1	Prostate adenocarcinoma	1.7% mutation and deletion on KIT only	1 of 59 patients	Tendency towards co-occurrence p=1.000	Michigan, Nature 2012
KIT PTEN	Prostate adenocarcinoma	1.3% mutation on KIT+PTEN	6 of 449 patients	Tendency towards mutual exclusivity p=0.989	CPC-GENE, Nature 2017
KIT PTEN	Prostate adenocarcinoma	19% mutation, deletion, and fusion on KIT+PTEN	86 of 448 patients	Tendency towards co-occurrence p=0.327	MSK, JCO Precision Oncology 2017
KIT PTEN	Prostate adenocarcinoma	43% mutation, amplification, deletion and fusion on KIT+PTEN	65 of 150 patients	Tendency towards mutual exclusivity p=0.074	Robinson et al., Cell 2015
KIT PTEN	Neuroendocrine prostate cancer	36% mutation, amplification, and deep deletion on KIT+PTEN	28 of 77 patients	Significant tendency towards co-occurrence p=0.010	Trento/Cornell/ Broad 2016

KIT PTEN	Prostate adenocarcinoma	20% mutation, amplification and deep deletion on KIT+PTEN	11 of 56 patients	Tendency towards co-occurrence p=0.328	Broad/Cornell, Cell 2013
KIT PTEN	Prostate adenocarcinoma	23% mutation, amplification, and deep deletion on KIT+PTEN	25 of 109 patients	Tendency towards mutual exclusivity p=0.607	Broad/ Cornell, Nat Genet 2012
KIT PTEN	Prostate adenocarcinoma	48% mutation, amplification and deep deletion on KIT+PTEN	26 of 54 patients	Tendency towards mutual exclusivity p=0.371	Fred Hutchinson CRC, Nat Med 2016
KIT PTEN	Prostate adenocarcinoma	17% mutation, amplification, and deep deletion on KIT+PTEN	17 of 103 patients	Tendency towards mutual exclusivity p=0.729	MSKCC, Cancer Cell 2010
KIT PTEN	Prostate adenocarcinoma	18% mutation, amplification, and deep deletion on KIT+PTEN	61 of 333 patients	Tendency towards co-occurrence p=0.537	TCGA, Cell 2015
KIT PTEN	Prostate adenocarcinoma	23% mutation, amplification, and deep deletion on KIT+PTEN	112 of 492 patients	Tendency towards mutual exclusivity p=0.534	TCGA, Provisional
KIT PTEN	Prostate adenocarcinoma	1.9% deep deletion on PTEN only	2 of 104 patients	Tendency towards co-occurrence p=1.000	MSKCC, PNAS 2014
KIT PTEN	Prostate adenocarcinoma	0% alterations	0 of 7 patients	Tendency towards co-occurrence p=1.000	MSKCC, Cell 2014
KIT PTEN	Prostate adenocarcinoma	51% mutation and deep deletion on KIT+PTEN	30 of 59 patients	Tendency towards co-occurrence p=0.492	Michigan, Nature 2012

KIT KRAS	Prostate adenocarcinoma	0.2% mutation on KIT only	1 of 449 patients	Tendency towards co-occurrence p=1.000	CPC-GENE, Nature 2017
KIT KRAS	Prostate adenocarcinoma	2% mutation, amplification, and deep deletion on KIT+KRAS	9 of 448 patients	Tendency towards mutual exclusivity p=0.968	MSK, JCO Precision Oncology 2017
KIT KRAS	Prostate adenocarcinoma	5% mutation and amplification on KIT+KRAS	8 of 150 patients	Tendency towards co-occurrence p=0.128	Robinson, et al., Cell 2015
KIT KRAS	Neuroendocrine prostate cancer	25% mutation and amplification on KIT+KRAS	19 of 77 patients	Significant tendency towards co-occurrence p<0.001	Trento/Cornell/ Broad 2016
KIT KRAS	Prostate adenocarcinoma	7% mutation and amplification on KIT+KRAS	4 of 56 patients	Tendency towards co-occurrence p=0.105	Broad/Cornell, Cell 2013
KIT KRAS	Prostate adenocarcinoma	17% amplification on KIT only	18 of 109 patients	Tendency towards co-occurrence p=1.000	Broad/Cornell, Nat Genet 2012
KIT KRAS	Prostate adenocarcinoma	7% mutation and amplification on KIT+KRAS	4 of 54 patients	Tendency towards mutual exclusivity p=0.942	Fred Hutchinson CRC, Nat Med 2016
KIT KRAS	Prostate adenocarcinoma	5% mutation, amplification and deep deletion on KIT+KRAS	5 of 103 patients	Tendency towards mutual exclusivity p=0.942	MSKCC, Cancer Cell 2010
KIT KRAS	Prostate adenocarcinoma	2.1% amplification and deep deletion on KIT+KRAS	7 of 333 patients	Tendency towards mutual exclusivity p=0.964	TCGA, Cell 2015

KIT KRAS	Prostate adenocarcinoma	2.4% mutation, amplification, and deep deletion on KIT+KRAS	12 of 492 patients	Tendency towards co-occurrence p=0.083	TCGA, Provisional
KIT KRAS	Prostate adenocarcinoma	0% alterations on KIT+KRAS	0 of 104 patients	Tendency towards co-occurrence p= 1.000	MSKCC, PNAS 2014
KIT KRAS	Prostate adenocarcinoma	0% alterations on KIT+KRAS	0 of 7 patients	Tendency towards co-occurrence p=1.000	MSKCC, Cell 2014
KIT KRAS	Prostate adenocarcinoma	8% mutation, amplification and deep deletion on KIT+KRAS	5 of 59 patients	Tendency towards mutual exclusivity p=0.934	Michigan, Nature 2012
KIT SRC	Prostate adenocarcinoma	0.2% mutation on KIT only	1 of 449 patients	Tendency towards co-occurrence p=1.000	CPC-GENE, Nature 2017
KIT SRC	Prostate adenocarcinoma	0.4% mutation on KIT only	2 of 448 patients	Tendency towards co-occurrence p=1.000	MSK, JCO Precision Oncology 2017
KIT SRC	Prostate adenocarcinoma	5% mutation, amplification and deep deletion on KIT+KRAS	7 of 150 patients	Tendency towards mutual exclusivity p=0.934	Robinson et al., Cell 2015
KIT SRC	Neuroendocrine prostate cancer	27% mutation and amplification on KIT+KRAS	21 of 77 patients	Significant tendency towards co-occurrence p=2.367	Trento/Cornell/ Broad 2016
KIT SRC	Prostate adenocarcinoma	4% mutation and amplification on KIT only	2 of 56 patients	Tendency towards co-occurrence p=1.000	Broad/Cornell, Cell 2013

KIT SRC	Prostate adenocarcinoma	17% mutation and amplification on KIT+SRC	18 of 109 patients	Tendency towards co-occurrence p=0.165	Broad/Cornell, Nat Genet 2012
KIT SRC	Prostate adenocarcinoma	11% mutation, amplification and deep deletion on KIT+SRC	6 of 54 patients	Tendency towards mutual exclusivity p=0.942	Fred Hutchinson CRC, Nat Med 2016
KIT SRC	Prostate adenocarcinoma	2.9% mutation and amplification on KIT+SRC	3 of 103 patients	Tendency towards mutual exclusivity p=0.981	MSKCC, Cancer Cell 2010
KIT SRC	Prostate adenocarcinoma	3% amplification and deep deletion on KIT+SRC	10 of 333 patients	Tendency towards mutual exclusivity p=0.930	TCGA, Cell 2015
KIT SRC	Prostate adenocarcinoma	2.4% mutation, amplification and deep deletion on KIT+SRC	12 of 492 patients	Tendency towards mutual exclusivity p=0.931	TCGA, Provisional
KIT SRC	Prostate adenocarcinoma	Genes not sequenced	0 of 104 patients	Tendency towards co-occurrence p=1.000	MSKCC, PNAS 2014
KIT SRC	Prostate adenocarcinoma	0% alterations	0 of 7 patients	Tendency towards co-occurrence p=1.000	MSKCC, Cell 2014
KIT SRC	Prostate adenocarcinoma	1.7% deep deletion on KIT only	1 of 59 patients	Tendency towards co-occurrence p=1.000	Michigan, Nature 2012

KIT WNT3	Prostate adenocarcinoma	0.2% mutation on KIT only	1 of 449 patients	Tendency towards co-occurrence p=1.000	CPC-GENE, Nature 2017
KIT WNT3	Prostate adenocarcinoma	0.4% mutation on KIT only; WNT3 not sequenced	2 of 448 patients	Tendency towards co-occurrence p=1.000	MSK, JCO Precision Oncology 2017
KIT WNT3	Prostate adenocarcinoma	4% mutation and amplification on KIT+WNT3	6 of 150 patients	Tendency towards mutual exclusivity p=0.967	Robinson et al., Cell 2015
KIT WNT3	Neuroendocrine prostate cancer	29% mutation and amplification on KIT+WNT3	22 of 77 patients	Significant tendency towards co-occurrence p<0.001	Trento/Cornell/ Broad 2016
KIT WNT3	Prostate adenocarcinoma	4% mutation and amplification on KIT only	2 of 56 patients	Tendency towards co-occurrence p=1.000	Broad/Cornell, Cell 2013
KIT WNT3	Prostate adenocarcinoma	17% mutation and amplification on KIT+WNT3	19 of 109 patients	Tendency towards mutual exclusivity p=0.835	Broad/Cornell, Nat Genet 2012
KIT WNT3	Prostate adenocarcinoma	9% mutation and amplification on KIT+WNT3	5 of 54 patients	Tendency towards mutual exclusivity p=0.928	Fred Hutchinson CRC, Nat Med 2016
KIT WNT3	Prostate adenocarcinoma	1.9% mutation and amplification on KIT only	2 of 103 patients	Tendency towards co-occurrence p=1.000	MSKCC, Cancer Cell 2010

KIT WNT3	Prostate adenocarcinoma	5% amplification and deep deletion on KIT+WNT3	16 of 333 patients	Tendency towards mutual exclusivity p=0.863	TCGA, Cell 2015
KIT WNT3	Prostate adenocarcinoma	4% mutation, amplification and deletion on KIT+WNT3	20 of 492 patients	Tendency towards mutual exclusivity p=0.828	TCGA, Provisional
KIT WNT3	Prostate adenocarcinoma	Genes not sequenced	0 of 104 patients	Tendency towards co-occurrence p=1.000	MSKCC, PNAS 2014
KIT WNT3	Prostate adenocarcinoma	0% alterations	0 of 7 patients	Tendency towards co-occurrence p=1.000	MSKCC, Cell 2014
KIT WNT3	Prostate adenocarcinoma	5% mutation and deep deletion on KIT+WNT3	3 of 59 patients	Tendency towards mutual exclusivity p=0.967	Michigan, Nature 2012
KIT WNT5A	Prostate adenocarcinoma	0.2% mutation on KIT only	1 of 449 patients	Tendency towards co-occurrence p=1.000	CPC-GENE, Nature 2017
KIT WNT5A	Prostate adenocarcinoma	0.4% mutation on KIT only; WNT5A not sequenced	2 of 448 patients	Tendency towards co-occurrence p=1.000	MSK, JCO Precision Oncology
KIT WNT5A	Prostate adenocarcinoma	4% mutation and amplification on KIT+WNT3	6 of 150 patients	Tendency towards mutual exclusivity p=0.967	Robinson et al., 2015
KIT WNT5A	Neuroendocrine prostate cancer	35% mutation and amplification on KIT+WNT3	27 of 77 patients	Significant tendency towards co-occurrence p=1.386	Trento/Cornell/ Broad 2016

KIT WNT5A	Prostate adenocarcinoma	4% mutation and amplification on KIT only	2 of 56 patients	Tendency towards co-occurrence p=1.000	Broad/Cornell, Cell 2013
KIT WNT5A	Prostate adenocarcinoma	17% amplification on KIT only	18 of 109 patients	Tendency towards co-occurrence p=1.000	Broad/Cornell, Nat Genet 2012
KIT WNT5A	Prostate adenocarcinoma	6% mutation, amplification and deep deletion on KIT+WNT5A	3 of 54 patients	Tendency towards mutual exclusivity p=0.956	Fred Hutchinson CRC, Nat Med 2016
KIT WNT5A	Prostate adenocarcinoma	1.9% mutation and amplification on KIT only	2 of 103 patients	Tendency towards co-occurrence p=1.000	MSKCC, Cancer Cell 2010
KIT WNT5A	Prostate adenocarcinoma	2.4% amplification and deep deletion on KIT+WNT5A	8 of 333 patients	Tendency towards mutual exclusivity p=0.953	TCGA, Cell 2015
KIT WNT5A	Prostate adenocarcinoma	2.8% mutation, amplification, and deep deletion on KIT+WNT5A	14 of 492 patients	Tendency towards mutual exclusivity p=0.904	TCGA, Provisional
KIT WNT5A	Prostate adenocarcinoma	Genes not sequenced	0 of 104 patients	Tendency towards co-occurrence p=1.000	MSKCC, PNAS 2014
KIT WNT5A	Prostate adenocarcinoma	0% alterations	0 of 7 patients	Tendency towards co-occurrence p=1.000	MSKCC, Cell 2014
KIT WNT5A	Prostate adenocarcinoma	3% mutation and deep deletion on KIT+WNT5A	2 of 59 patients	Tendency towards mutual exclusivity p=0.984	Michigan. Nature 2012

KIT NOTCH1	Prostate adenocarcinoma	0.2% mutation on KIT only	1 of 449 patients	Tendency towards co-occurrence p=1.000	CPC-GENE, Nature 2017
KIT NOTCH1	Prostate adenocarcinoma	2.5% mutation and deep deletion on KIT+NOTCH1	11 of 448 patients	Tendency towards mutual exclusivity p=0.953	MSK, JCO Precision Oncology 2017
KIT NOTCH1	Prostate adenocarcinoma	6% mutation and amplification on KIT+NOTCH1	9 of 150 patients	Tendency towards co-occurrence p=0.158	Robinson et al., Cell 2015
KIT NOTCH1	Neuroendocrine prostate cancer	32% mutation and amplification on KIT+NOTCH1	25 of 77 patients	Significant tendency towards co-occurrence p<0.001	Trento/Cornell/ Broad 2016
KIT NOTCH1	Prostate adenocarcinoma	5% mutation and amplification on KIT+NOTCH1	3 of 56 patients	Tendency towards mutual exclusivity p=0.964	Broad/Cornell, Cell 2013
KIT NOTCH1	Prostate adenocarcinoma	17% amplification on KIT only	18 of 109 patients	Tendency towards co-occurrence p=1.000	Broad/Cornell, Nat Genet 2012
KIT NOTCH1	Prostate adenocarcinoma	17% mutation and amplification on KIT+NOTCH1	9 of 54 patients	Tendency towards co-occurrence p=0.183	Fred Hutchinson CRC. Nat Med 2016
KIT NOTCH1	Prostate adenocarcinoma	4% mutation, amplification and deep deletion on KIT+NOTCH1	4 of 103 patients	Tendency towards mutual exclusivity p=0.961	MSKCC, Cancer Cell 2010
KIT NOTCH1	Prostate adenocarcinoma	2.7% amplification and deep deletion on KIT+NOTCH1	9 of 333 patients	Tendency towards mutual exclusivity p=0.941	TCGA, Cell 2015

KIT NOTCH1	Prostate adenocarcinoma	3% mutation, amplification and deep deletion on KIT+NOTCH1	16 of 492 patients	Tendency towards co-occurrence p=0.135	TCGA, Provisional
KIT NOTCH1	Prostate adenocarcinoma	Genes not sequenced	0 of 104 patients	Tendency towards co-occurrence p=1.000	MSKCC, PNAS 2014
KIT NOTCH1	Prostate adenocarcinoma	0% alterations	0 of 7 patients	Tendency towards co-occurrence p=1.000	MSKCC, Cell 2014
KIT NOTCH1	Prostate adenocarcinoma	7% mutation, amplification, and deep deletion on KIT+NOTCH1	4 of 59 patients	Tendency towards mutual exclusivity p=0.951	Michigan, Nature 2012

Table S3. Mutations and Amplifications of CD117/KIT and Associated Signaling Pathways in Cancer Datasets. Using cBioPortal the percentage of amplifications, mutations, deletions, and alterations were mined for the *KIT* gene and signaling genes for prostate adenocarcinoma. Summarized data is shown in the table.
